# The predominance of recombinant Norovirus GII.4Sydney[P16] strains in children less than 5 years of age with acute gastroenteritis in Tehran, Iran, 2021–2022

**DOI:** 10.1016/j.virusres.2023.199172

**Published:** 2023-07-24

**Authors:** Mahtab Eftekhari, Atefeh Kachooei, Somayeh Jalilvand, Tayebeh Latifi, Zahra Habib, Angila Ataei-Pirkoohi, Sayed Mahdi Marashi, Zabihollah Shoja

**Affiliations:** a Department of Virology, School of Public Health, Tehran University of Medical Sciences, Tehran, Iran; b Department of Virology, School of Medicine, Iran University of Medical Sciences, Tehran, Iran; c Department of Virology, Pasteur Institute of Iran, Tehran, Iran; d Research Center for Emerging and Reemerging Infectious Diseases, Pasteur Institute of Iran, Tehran, Iran

**Keywords:** Norovirus, Acute gastroenteritis, Dual-typing, RdRp, VP1

## Abstract

The present study was aimed to both detect emerging noroviruses and investigate RdRp and VP1-based dual typing of circulating noroviruses in hospitalized children less than 5 years of age with acute gastroenteritis (AGE) in Iran. For this purpose, a total of 200 stool specimens were screened during 2021–2022 by real-time RT-PCR for genogroup I and II (GI and GII) and dual-typed by sequence analysis of PCR products, using a web-based norovirus Typing Tool and phylogenetic analysis. The GI and GII noroviruses were detected in 20% of 200 specimens. The GII.4 norovirus was found to be the most common VP1 genotype (53%) followed by GII.8 (32%), GII.7 (6%), GII.17 (6%), and GII.3 (3%). The GII.P16 norovirus was also found as the predominant RdRp type (53%) followed by GII.P8 (32%), GII.P7 (6%), GII.P17 (6%), and GII.P31 (3%). To our knowledge, this is the first report that highlights the dominancy of recombinant norovirus GII.4Sydney[P16] and newly emerging of norovirus GII.8 [P8], GII.17 [P17] and GII.3 [P16] in Iran. These findings further indicate inter-genotype recombinant strains of noroviruses.

## Highlights

• The RdRp and VP1-based dual typing by genetic analysis of circulating NoVs in Iran.
• GII.4 Sydney_2012[P16] was determined as the most common strain.
• GII.8[P8] viruses were also found to be as the second most common.

## 1 Introduction

Noroviruses are the most common cause of both sporadic and epidemic non-bacterial acute gastroenteritis (AGE) across all age groups. Moreover, noroviruses are the second leading cause of AGE in children less than 5 years of age, after rotavirus, associated with an estimated 70,000–200,000 deaths annually, mostly in developing countries (Pires et al., 2015; Cannon et al., 2019; Bányai et al., 2018). Noroviruses have also emerged and well recognized as the predominant pediatric viral enteric agent following the implementation of rotavirus vaccine. Regarding the global prevalence of noroviruses, several systematic review and meta-analysis have indicated that norovirus prevalence increased from 12% in 1990 −2008 (Patel et al., 2008) to 18% in 2015–2021 (Ahmed et al., 2014; Liao et al., 2021; Farahmand et al., 2022), which further highlights the increasing trend of norovirus infections.

In 1979, Kapikian and others were first identified *Norwalk* virus, the prototype agent of the genus norovirus (previously denoted as “*Norwalk-like viruses*”), as the etiological cause of AGE, during an outbreak of gastroenteritis in students at an elementary school in Norwalk, Ohio, United States (Kapikian et al., 1972). Noroviruses, as a genus of the *Caliciviridae* family, are non-enveloped, positive sense with a single-stranded RNA genome 7.5–7.7 kb in length (Chhabra et al., 2019). The genome is organized into three open reading frames (ORFs) with ORF1 encoding the nonstructural (NS) viral proteins, ORF2 and ORF3 encoding the major (VP1) and minor (VP2) capsid proteins, respectively (Chhabra et al., 2019). Complete VP1 amino acid sequences and the ORF1 NS7 region (which encodes the RNA-dependent RNA polymerase; RdRp) nucleotide sequences were used for classification based on its genetic variability (Chhabra et al., 2019). Accordingly, based on the complete amino acid sequences of VP1 gene, 10 genogroups (G1-GX) of norovirus have been established so far. Among which, norovirus GI, GII, GIV, GVIII, and GIX are reported to infect humans although GI and GII is by far the most medically important species globally (Chhabra et al., 2019). These genogroups were further segregated into 49 confirmed genotypes, based on amino acids of the complete VP1. Among genotypes, GII genotype 4 (GII.4) is the most predominantly reported circulating strain globally, and can be classified into subtypes or variants. Every 2 to 3 years, new GII.4 variants emerge and replace the previously predominant strains (Chhabra et al., 2019; Kroneman et al., 2013). In 1995, GII.4 pandemic first documented by GII.4 US95_96 variant, followed by the emergence of GII.4 Farmington Hills in 2002, GII.4 Hunter in 2004, GII.4 Yerseke and GII.4 Den Haag in 2006, GII.4 New Orleans in 2009, and GII.4 Sydney in 2012 (Kroneman et al., 2013; Vinjé, 2015; Tohma et al., 2019) as well as several local epidemics of GII.4 variants such as Grimsby 1995, Henry 2001, Japan 2001, Asia 2003, 2006a, Cairo 2007, Apeldoorn 2008, and Japan 2008 (Tohma et al., 2019; Tu et al., 2008; Parker et al., 2005; Seto et al., 2005; Belliot et al., 2010; Kamel et al., 2009; Motomura et al., 2010). Recombination, as a major driving force of norovirus diversity, may further increased the emergence of novel strains (Chhabra et al., 2019). The recombination breakpoints are most frequently identified in the ORF1-ORF2 junction region (Chhabra et al., 2019). In this regard, dual typing has been proposed to include diversity at the level of the partial RdRp sequences in strain designations. Based on nucleotide diversity in the RdRp region, noroviruses can be classified into 60 P-types (Chhabra et al., 2019). Based on the last norovirus classification update, it has been proposed the following designations of norovirus strains: GI.genotype[P-type] or GII.genotype[P-*type*], first listing the capsid genotype followed by the P-type between brackets (Chhabra et al., 2019). In this regard, Kendra et al., (Kendra et al., 2022) recently analyzed dataset of norovirus sequences between the years 1995–2019 and documented that GII.4Sydney[P31], GII.2[P16], GII.4SydneyP[16] recombinant strains associated with more than 50% of all AGE outbreaks due to noroviruses (Kendra et al., 2022).

Previous studies in Iran have indicated that positivity rates of norovirus in children less than 5 years of age ranges from 4.14 to 21.3% (Fazeli et al., 2010; Jalilian et al., 2012; Najafi et al., 2013; Romani et al., 2012; Roodsari et al., 2013; Sharifi-Rad et al., 2015; Shoja et al., 2014; Nasab et al., 2016). Although little information is available with regards to the norovirus genotypes in Iran, GII.4 is shown to be the most predominant capsid genotype, followed by GII.3, GII.2, GII.6, GII.7, GII.12, GI.4 (Romani et al., 2012; Farsi et al., 2018). While studies on noroviruses were mainly limited to the prevalence of noroviruses, less data are available regarding RdRp and VP1-based dual typing of noroviruses circulating in Iran. As such, the present study was aimed to identify the genetic variation and dual typing pattern of norovirus strains across the Iranian children on the basis of distinct RdRp and VP1 genes.

## 2 Materials and methods

### 2.1 Specimen collection

Fecal specimens were collected from children less than 5 years of age with AGE at Children's Hospitals in Tehran from January 2021 to January 2022. Specimens were transported and stored at – 20 °C in the laboratory of Molecular Virology Division at Pasteur Institute of Iran. The study was conducted according to the Helsinki guidelines and approved by the ethics committee of the Pasteur Institute of Iran.

### 2.2 Viral RNA extraction and cDNA synthesis

10% (wt/vol) fecal suspension was prepared with phosphate-buffered saline (PBS) and clarified using centrifugation at 1500 x g for 20 min. Viral RNA was then extracted from clarified supernatant using viral RNA/DNA extraction kit (GeneAll, Korea) according to the manufacturer's instructions. Extracted RNA was used for complementary DNA (cDNA) synthesis, which was performed with SinaClon First Strand cDNA synthesis kit (Cat. No. RT5201; SINACLON, Tehran, Iran) using a single cycle in a final volume of 20 μL according to the manufacturer's instructions.

### 2.3 Screening of GI and GII noroviruses

The presence of GI and GII noroviruses in fecal specimens was assessed using real-time PCR with the Corbett Research Rotor-GeneTM (Qiagen, USA). The primer and probe sets COG1F, COG1R, probe Ring 1 and COG2F, COG2R, probe Ring 2 (Table 1) were used to screen GI and GII norovirus strains, respectively (Kageyama et al., 2003; Rolfe et al., 2007). Briefly, PCR reactions were prepared in 20 μl reaction mixtures containing cDNA, 4X CAPITAL™ qPCR Probe Master Mix (BiotechRabbit, Germany), 500 nM of each primer, and 100 nM of each probe. PCR amplification were performed at 95°C for 2–3 min, followed by 40 cycles of PCR at 95°C for 5 s and 60°C for 30 s.

**Table 1:** List of primers used in this study.

Purpose	Primer/prob name	Sequence 5′ to 3′	Genogroup	References	
Screening of GI and GII NoVs	COG1F	CGYTGGATGCGNTTYCATGA	GI	Kageyama et al., 2003, Rolfe et al., 2007	
	COG1R	CTTAGACGCCATCATCATTYAC			
	Ring 1	FAM-AGATYGCGATCYCCTGTCCA-TAMRA			
	COG2F	CARGARBCNATGTTYAGRTGGATGAG	GII		
	COG2R	TCGACGCCATCTTCATTCACA			
	Ring 2	FAM-TGGGAGGGCGATCGCAATCT-BHQ			
RdRP typing	First round	JV12Y	ATA CCA CTA TGA TGC AGA YTA	typing GI and GII-by-sequencing	Vennema et al., 2002
		COG2R	TCG ACG CCA TCT TCA TTC ACA		
	Secound round	JV12Y	ATA CCA CTA TGA TGC AGA YTA		
		JV13I	TCA TCA TCA CCA TAG AAI GAG		
VP1 genotyping	First round	COG2F	CAR GAR BCN ATG TTY AGR TGG ATG AG	Genotyping GII-by-sequencing	Kojima et al., 2002
		G2SKR	CCR CCN GCA TRH CCR TTR TAC AT		
	Secound round	G2SKF	CNT GGG AGG GCG ATC GCA A		
		G2SKR	CCR CCN GCA TRH CCR TTR TAC AT		
	First round	COG1F	CGYTGGATGCGNTTYCATGA	Genotyping GI-by-sequencing	
		G1SKR	CCAACC CAR CCA TTR TAC A		
	Secound round	G1SKF	CTG CCC GAA TTY GTA AAT GA		
		G1SKR	CCAACC CAR CCA TTR TAC A		

Degenerate Nucleotide Codes: B = C/G/T; *H* = A*/*C/T; *N* = A*/*C/G/T; *R* = A*/*G; Y = C/T; *W* = A*/*T.

### 2.4 Amplification of partial RDRP and VP1 of GI and GII noroviruses

The positive specimens by real-time RT-PCR were then genotyped for the RdRp and VP1 regions. Semi-nested PCR was used to amplify RdRp and VP1 regions using primer sets COG2R, JV12Y, JV13I (Vennema et al., 2002) (for GI and GII) and COG1F, G1SKF, G1SKR (Kojima et al., 2002) (for GI), COG2F, G2SKF, G2SKR (Kojima et al., 2002) (for GII) (Table 1). The PCR amplification reactions for the first round PCR were prepared in 25 μl reaction mixtures containing 2X Hot-Start PCR Master Mix (BiotechRabbit, Germany), 500 nM of each first round primers (JV12Y/COG2R for RdRp; COG1F/G1SKR and COG2F/G2SKR for VP1 of GI and GII, respectively), and cDNA as template. The nested-PCR amplification reactions were also performed in 25 μl reaction mixtures containing 2X Hot-Start PCR Master Mix (BiotechRabbit, Germany), 500 nM of each second primers (JV12Y/JV13I) for RdRp; G1SKF/G1SKR and G2SKF/G2SKR for VP1 of GI and GII, respectively), and 1 μl of the first round PCR product. The following condition was applied for PCR amplification of both rounds: initial activation for 2 min at 95 °C, followed by 35 cycles of denaturation for 30 s at 95 °C, annealing for 30 s at 37 °C, extension for 45 s at 72 °C, and a final extension for 5 min at 72 °C. The PCR products of the expected size were finally visualized in 1.5% agarose gel and sequenced.

### 2.5 Sequence analysis and molecular typing

The nucleotide sequence of the partial RdRp and VP1 was obtained by sequencing with a 3130 Genetic Analyzer Automated Sequencer following Applied Biosystems protocols (Applied BioSystems, Foster City, CA, USA) and edited with the CLC Main Workbench (CLC Bio). Sequences were then genotyped using a web-based Norovirus Typing Tool Version 2 (Tatusov et al., 2021). Multiple sequence alignments of norovirus RdRp and VP1 nucleotide sequences of Iran as well as the reference norovirus strains from GenBank were established using BioEdit. The Clustal W method was used to generate phylogenetic tree using the maximum likelihood method (MLM) based on the Kimura 2-parameter model (MEGA 6 software) (Tamura et al., 2013). The trees were statistically supported by bootstrapping with 1000 replicates.

## 3 Results

### 3.1 Detection and genetic analysis of norovirus

**Fig. 1:**
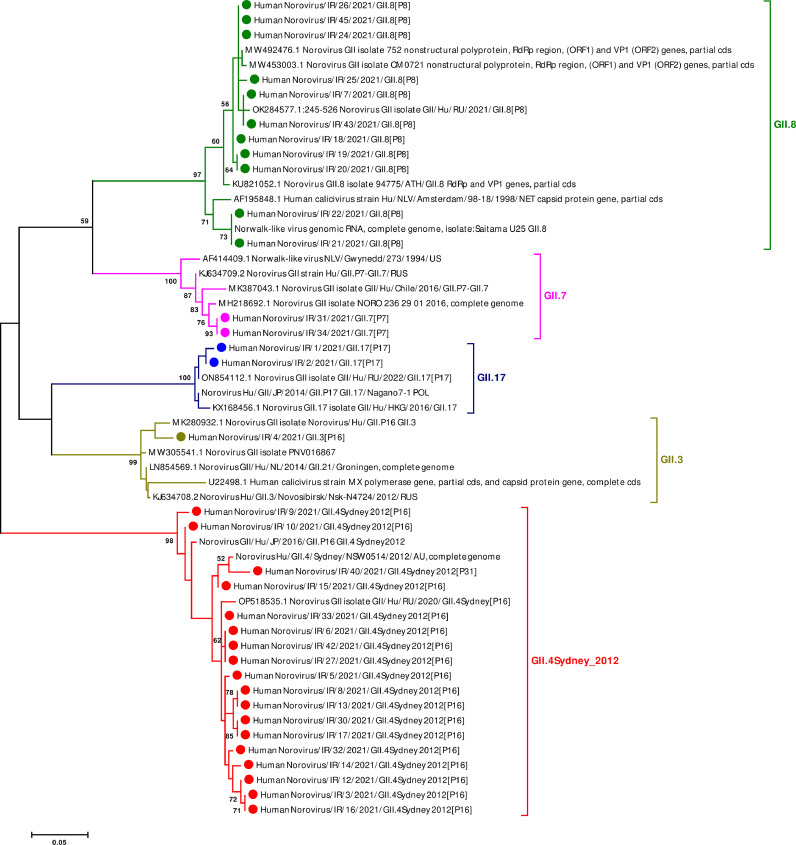

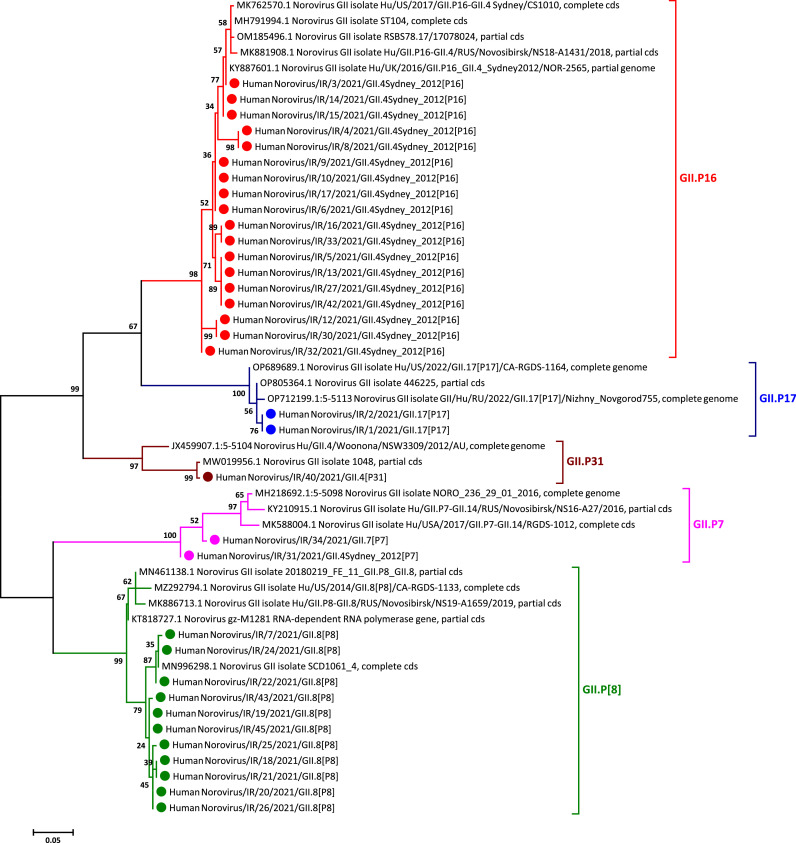
Phylogenetic analysis based on the partial nucleotide sequence of the VP1 (A) and RdRp gene (B). The phylogenetic trees were generated by using the Maximum Likelihood method (MLM) based on the Kimura 2-parameter model, with 1000 bootstrap replications for branch support. Only bootstrap values greater than 50% are presented. The tree was drawn to scale with the bar at the bottom indicating 0.05 nucleotide substitutions per site. The norovirus GII strains detected in this study are indicated in solid circle.

Among 200 feces specimens tested, 20% (40 out of 200) were positive for GI and GII noroviruses by real-time RT-PCR. All norovirus strains were obtained directly from the fecal specimens without virus multiplication in cell culture. Positive noroviruses were considered for their VP1 genotypes and RdRp P typing. The VP1 and RdRP nucleotide sequences with no ambiguous positions and heterogeneity were determined and investigated using automated norovirus genotyping tool and phylogenetic analysis. The majority of noroviruses (95%; 38 out of 40) was found to be norovirus GII and only 5% (2 out of 40) norovirus GI. In overall, 35 samples (34 in GII and 1 in GI) were successfully sequenced for the partial RdRp and VP1 genes and 5 sample (4 in GII and 1 in GI) failed. The phylogenetic analyses for VP1 indicated that the VP1 sequences identified in Iran belongs to GII.3, GII.4, GII.7, GII.8, and GII.17 clusters, as statistically supported by 97–100% bootstrap value (Fig. 1A). We found five VP1 genotypes of norovirus GII, including GII.3 (3%, *n* = 1), GII.4 (53%, *n* = 18), GII.7 (6%, *n* = 2), GII.8 (32%, *n* = 11), GII.17 (6%, *n* = 2). In the present study, the norovirus GII.4 viruses were the most prevalent VP1 genotypes, accounting for more than 50% of all strains, which belonged to variant GII.4 Sydney_2012 (Fig. 1A). Moreover, phylogenetic analyses of RdRp indicated that the RdRp sequences identified in Iran belongs to GII.P7, GII.P8, GII.P16, GII.P17, and GII.P31 clusters, as statistically supported by 97–100% bootstrap value (Fig. 1B). Five P types of norovirus GII were also detected, including GII.P7 (6%, *n* = 2), GII.P8 (32%, *n* = 11), GII.P16 (53%, *n* = 18), GII.P17 (6%, *n* = 2), and GII.P31 (3%, *n* = 1). According to our findings, norovirus GII.P16 viruses were found to be the most prevalent P types, accounting for more than 50% of all strains. Based on dual typing analysis using automated norovirus genotyping tool and phylogenetic analysis, the 34 norovirus GII strains were further classified into six different VP1-RdRp combinations: GII.4 Sydney[P16] (50%, *n* = 17), GII.4Sydney[P31] (3%, *n* = 1), GII.3[P16] (3%, *n* = 1), GII.7[P7] (6%, *n* = 2), GII.8[P8] (32%, *n* = 11), and GII.17[P17] (6%, *n* = 2) (Table 2). Discordant RdRp and VP1 genotypes that were identified in 19 of 34 norovirus strains including GII.4Sydney[P16] (50%, *n* = 17), GII.4Sydney[P31] (3%, *n* = 1), and GII.3[P16] (3%, *n* = 1), considered GII.4Sydney[P16] as a most dominant virus, further indicating inter-genotype recombinant strains. The only norovirus GI typed in this study was GI.3 VP1 genotype, which was related to GI.P3 RdRp type (GI.3[P3]).

**Table 2:** Distribution of GII NoV strains (Dual typing) in stool specimens of hospitalized children < 5 years of age with AGE in Iran, 2021–2022.

n (%)	VP1/RdRP dual typing
17 (50)	GII.4 Sydney_2012[P16]
11 (32)	GII.8[P8]
2 (6)	GII.7[P7]
2 (6)	GII.17[P17]
1 (3)	GII.3[P16]
1 (3)	GII.4 Sydney_2012[P31]
34 (100)*	Total

⁎ Overall 35 samples (34 in NoV GII and 1 in NoV GI) were successfully sequenced for the partial RdRp and VP1 genes and values in this table indicate the percentage of the total of 34 of NoV GII.

## 4 Discussion

Norovirus infections in children with AGE, which has been reported from 2006 to 2014 from several regions of Iran, well indicate the prevalence rates of norovirus infection from 4.14 to 21.3% (Fazeli et al., 2010; Jalilian et al., 2012; Najafi et al., 2013; Romani et al., 2012; Roodsari et al., 2013; Sharifi-Rad et al., 2015; Shoja et al., 2014; Nasab et al., 2016). In our previous study, we have detected genogroup GII of norovirus in 17.1% of specimens taken from children less than 5 years of age with AGE (Farsi et al., 2018). The overall prevalence of norovirus infection in the present study was found to be 20% in children with AGE, which was higher than the previous report from Iran. The GII viruses were significantly predominant, representing 95% of the total norovirus infections. In addition to detection and analysis of norovirus GI and GII prevalence, we also investigated the genetic characterization of noroviruses in the community, which further highlighted the circulation and dominancy of recombinant norovirus GII.4 Sydney[P16] in Iran. Furthermore, we detected newly emerging of norovirus GII.8[P8], GII.17[P17] and GII.3[P16], and also the formerly GII.7[P7], GII.3[P3] and GII.4 Sydney[P31] strains. The genogroup GI of norovirus accounted for 5% of all norovirus infections, which one sample were successfully sequenced for the partial RdRp and VP1 genes and typed as GI.3[P3]. The GI.3 genotype was reported to be the most frequently detected GI viruses in outbreaks in China, Republic of Korea, Tailand, and Taiwan that mainly occurred in pre-school and school students (Chiu et al., 2020).

As the most prevalent strains, GII.4 variants have been reported worldwide, which related to GII.P4 and GII.P31 (P31 previously known as GII.Pe) RdRp sequences (Matsushima et al., 2016). The recombinant norovirus GII.4Sydney[P16] was first emerged in 2016 at a low prevalence (Matsushima et al., 2016; J.H. Lun et al., 2018; Choi et al., 2017; Niendorf et al., 2017). GII.4Sydney[P16], as the most latest recombinant norovirus strain derived from the previous GII.4Sydney_2012 variant, harboring a new discordant RdRp type P16. Since then and as result of different recombination events, GII.P16 noroviruses have been identified in combination with several capsid types, including GII.1, GII.2, GII.3, GII.10 and GII.12 (Bonura et al., 2021). In our present and previous studies (Farsi et al., 2018), we detected GII.P16 noroviruses, which related to several capsid types such as GII.3, GII.4, and GII.12. In 2018, we have reported that recombinant norovirus GII.4Sydney[P31] was predominant in specimens obtained Iranian children from 2015 to 2016, concomitant with the emergence of two novel GII.4 recombinant viruses, GII.4Sydney[P16], and GII.4Sydney[P2] both of which retained the Sydney 2012 capsid but acquired new discordant RdRp (Farsi et al., 2018). Our current findings indicate a switch in strain predominance, where recombinant norovirus GII.4Sydney[P16], as the most dominant strain, replaced the previously dominant GII.4Sydney[P31] in Iran. To support this, several previous studies have shown that the new recombinant GII.4_Sydney[P16] strain emerged in 2016, replacing GII.4_Sydney[P31] in some countries (Barclay et al., 2019; J.H. Lun et al., 2018; Jones et al., 2018; Barreira et al., 2017). Indeed, similar trend in the current recombinant GII.4_Sydney[P16] circulation has been observed in several countries, such as the Brazil, South Korea, USA, Japan, France, and England (Matsushima et al., 2016; Choi et al., 2017; Barreira et al., 2017; Cannon et al., 2017; Ruis et al., 2017; Bidalot et al., 2017).

GII.8[P8] viruses were also found to be as the second most common virus after recombinant GII.4_Sydney[P16] in children less than 5 years of age with AGE, which have not been reported previously in Iran. The GII.8P[8] belongs to a non-epidemic strains that was often identified with low positive rates in several regions and less common in children (Bok et al., 2009; Mori et al., 2017). The association of GII.8[P8] with AGE outbreaks has been previously reported in restaurants and hotels (Lysén et al., 2009) and also in monitoring on water and shellfish (Nishida et al., 2003). Also less common, in the present study we found the formerly emergent GII.3[P3] and GII.7[P7] as well as new emergent GII.17[P.17] viruses. It has been shown that non-GII.4 including, GII.2, GII.3, and GII.17 viruses can also surprisingly become prevalent (Tohma et al., 2017). In the studies available from 1970s to 2000s, the GII.3 strain, instead of GII.4, has been documented to be the most prevalent genotype in children with AGE (Boon et al., 2011; Mahar et al., 2013), which related to GII.P3 and/or non-GII.3 RdRp types such as GII.P4, GII. P12, GII.P16, GII.P21, GII.P22, GII.P29 and GII.P31. Among which, GII.3[P21] has been found more frequently, which was firstly reported in 2002 in Spain during an outbreak and sporadic cases with AGE (Buesa et al., 2002). In Iran, the GII.3 emergent virus, as the most common strain, had previously been detected in specimens obtained from Iranian children from 2008 to 2009 (Romani et al., 2012) although its P typing of RdRp does not determined. Furthermore, we previously detected recombinant GII.3 in five specimens, which harbored the GII.P21 RdRp (Farsi et al., 2018), which this RdRp discordancy for GII.3 in present study was reported for one sample with combination of GII.3[P16].

In conclusion, the present study indicated yearly diversity in the predominant norovirus strains and/or recombinants compared to our previous study. To our knowledge, this is the first report that highlights the dominancy of recombinant norovirus GII.4 Sydney[P16] and newly emerging of norovirus GII.8[P8], GII.17[P17] and GII.3[P16]. In line with changing the global pattern of norovirus strains distribution, circulating norovirus strains in Iran may also change as indicated by our findings. Indeed, although GII.4Sydney[P31] has been a predominant strain during 2015–2016, it was replaced by GII.4Sydney[P16] and GII.8[P8] in 2021–2022. Our findings indicate that new and recombinant norovirus strains emerged in Iran and circulating strains can changes over time.

## CRediT authorship contribution statement

Mahtab Eftekhari, Atefeh Kachooei: Specimens collection, methodology, investigation, visualization. Zahra Habib: Specimens collection and preparation. Tayebeh Latifi and Angila Ataei-Pirkooh: Investigation and data analysis. Somayeh Jalilvand, and Sayed Mahdi Marashi: Supervision, writing-review, and editing. Zabihollah Shoja: conceptualization, supervision, methodology, visualization, and writing-original draft.

## Declaration of Competing Interest

The authors declare that they have no known competing financial interests or personal relationships that could have appeared to influence the work reported in this paper.

## Data Availability

Data will be made available on request.
